# Economic burden of adult patients with β-thalassaemia major in mainland China

**DOI:** 10.1186/s13023-023-02858-4

**Published:** 2023-08-29

**Authors:** Xuemei Zhen, Jing Ming, Runqi Zhang, Shuo Zhang, Jing Xie, Baoguo Liu, Zijing Wang, Xiaojie Sun, Lizheng Shi

**Affiliations:** 1 Centre for Health Management and Policy Research, School of Public Health, Cheeloo College of Medicine, Shandong University, No.44 Wenhuaxi Road, Jinan, 250012 Shandong China; 2 NHC Key Lab of Health Economics and Policy Research, Shandong University), No.44 Wenhuaxi Road, Jinan, 250012 Shandong China; 3 Beijing New Sunshine Charity Foundation, No.25 Landianchangnan Road, Beijing, 100097 China; 4 Department of Health Policy and Management, School of Public Health and Tropical Medicine, Tulane University, New Orleans, LA 70112 USA

**Keywords:** Economic burden, Lifetime cost, Adult patient, β-thalassaemia major, China

## Abstract

**Background:**

β-thalassaemia major poses a substantial economic burden, especially in adults. We aimed to estimate the economic burden of adult patients with β-thalassaemia major from a societal perspective using the real-world data. According to the clinical guideline, we also estimated the annual medical costs for patients with the same body weight and calculated the lifetime medical costs over 50 years in mainland China.

**Methods:**

This was a retrospective cross-sectional study. An online survey with snowball sampling covering seven provinces was conducted. We extracted patient demographics, caregiver demographics, disease and therapy information, caring burden, and costs for adult patients diagnosed with β-thalassaemia major and their primary caregivers. In the real world, we estimated the annual direct medical cost, direct nonmedical cost, and indirect cost. In addition, we calculated the annual direct medical cost and lifetime direct medical cost by weight with discounted and undiscounted rates according to the clinical guideline.

**Results:**

Direct medical costs was the main driver of total cost, with blood transfusion and iron chelation therapy as the most expensive components of direct medical cost. In addition, adult patients with β-thalassaemia major weighing 56 kg were associated with an increase of $2,764 in the annual direct medical cost using the real-world data. The undiscounted and discounted (5% discount rate) total lifetime treatment costs were $518,871 and $163,441, respectively.

**Conclusions:**

Patients with β-thalassaemia major often encounter a substantial economic burden in mainland China. Efforts must be made to help policymakers develop effective strategies to reduce the burden and pevalence of thalassaemia.

**Supplementary Information:**

The online version contains supplementary material available at 10.1186/s13023-023-02858-4.

## Introduction

β-thalassaemia is a global and regional disease, most highly prevalent in the Mediterranean, Middle East, central Asia, India, and Southern China. The nationwide prevalence of β-thalassaemia is 0.66% in mainland China and as high as 2.21% in high-incidence provinces (such as Guangdong and Guangxi) [1]. β-thalassaemia major, the most severe form, requires regular lifelong blood transfusions and chelation therapy [2]; and poor compliance with blood transfusions and chelation therapy leads to the risks of anaemia and iron overload, resulting in organ damage and complications [3].

Over the last three decades, most patients with β-thalassaemia major were unable to survive to adulthood, mainly because conventional blood transfusions and iron chelation therapy were too burdensome for full adherence. For example, approximately 80% of United Kingdom (UK) patients with β-thalassaemia major died before the age of 45 years [4]. One study with 1,029 patients revealed that adult patients with β-thalassaemia major were rare, and only 3% of patients were 18 years or older in Guangxi,China [5]. In addition, progressive disease results in multiple organ dysfunction, chronic pain, loss of physical fitness, deteriorating quality of life into adulthood, and more challenges than in childhood (such as working and marriage) [6].

The financial burden increases as patients aged and gained weight. Long-term treatment for patients with β-thalassaemia major poses a substantial economic burden on the health care system, patients, and their families [7]. Studies on the economic burden of β-thalassaemia major have been reported in the UK, United States (US), Italy, Iran, Thailand, Taiwan, and India, mainly focusing on children [8–15]. The annual treatment costs of β-thalassaemia major varied across countries and ranged from $950 to $128,062 [8, 9, 11, 15]. This massive economic burden is primarily attributed to direct medical cost, and blood transfusions and iron chelation therapy are the most expensive components of direct medical costs [16]. In addition, higher direct medical costs have been found in adult patients than in child patients [17].

Although the direct medical cost in real world has been quite heavy, many patients in China are experiencing undertreatment, and they haven’t received enough blood transfusion and iron chelation owing to poor compliance with the clinical guideline, compared to those in developing countries [2]. Therefore, the economic burden estimation approach based on the real world data may underestimate the medical cost of regular treatments recommended by the clinical guideline. To our knowledge, there is no study of β-thalassaemia major employing adult patient-specific cost data including both direct and indirect costs from a societal perspective, and there is a gap in estimating the lifetime costs for patients with β-thalassaemia major according to the clinical guideline in mainland China.

In this study, we aimed to (1) estimate the economic burden of adult patients with β-thalassaemia major from a societal perspective in the real world, including direct medical costs, direct nonmedical costs, and indirect costs, and (2) estimate the annual medical costs for patients with the same weight and calculate the lifetime medical costs over 50 years, according to the clinical guideline.

## Methods

### Study design and participants

This is a retrospective cross-sectional study. An online survey with the “questionnaire star (https://www.wjx.cn)” was conducted between September 1, 2021, and January 31, 2022, because an on-site survey was not feasible during the coronavirus disease 2019 (COVID-19) pandemic. Patients were recruited through the website of the Beijing New Sunshine Charity Foundation and the Thalassaemia Mutual Aid WeChat Group, and patient recommendations by doctors from representative medical institutions in five provinces (Guangdong Province, Guangxi Zhuang Autonomous Region, Yunnan Province, Hainan Province, and Fujian Province) were used as well. Snowball sampling was selected in this study because of the following reasons: first,the prevalence of thalassaemia in different provinces was unclear; second, enough participants were difficult to find, because the patients’ visiting medical institution was not fixed due to the unstable supply of blood and iron chelation, as well as lifetime or irregular therapy; and third, patient’s complete medical cost information were not possible to be collected in one medical institution. In addition, to ensure that there were no missing items, logical errors or irregularities in completion, two strict quality control interviews was conducted via telephone. If necessary, the patients were asked to show hospital expense invoices to provide relatively accurate expense information.

β-thalassaemia major can be diagnosed using blood tests (including complete blood counts and special haemoglobin tests) and genetic testing (e.g. DNA testing) [18]. Adult patients diagnosed with β-thalassaemia major and their caregivers were included in this study. The inclusion criteria for patients were as follows: (a) age ≥ 18 years old; (b) a diagnosis of β-thalassaemia major before the study; (c) understanding the content of the questionnaire; (d) a primary caregiver who was familiar with the entire treatment process; and (e) completion of two quality control interviews.

### Data collection

The questionnaire, which included a patient section and a caregiver section, was self-reported by eligible adult patients with β-thalassaemia major and their caregivers. We extracted patient demographics (province, sex, age, ethnicity, marital status, employment status, weight, daily wages), caregiver demographics (sex, age, ethnicity, marital status, employment status, education, identity, daily wage), disease and therapy information (comorbidity, disease therapy duration, pretransfusion haemoglobin level, interruption of blood transfusion therapy or iron chelation therapy, total days of lost wages annually), caring burden (total days of lost wages annually), and costs (direct medical cost, direct nonmedical cost).

Direct medical costs, were calculated by patient weight according to the clinical guideline for the diagnosis and treatment of β-thalassaemia major, which was published by the Subspecialty Group of Haematology, the Society of Paediatrics, and the Chinese Medical Association combined with the Editorial Board, Chinese Journal of Paediatrics in 2017 [18], aiming to improve the quality of care through the standardization of management based on evidence in published literature and expert opinion.

According to this clinical guideline, patients with β-thalassaemia major are recommended to receive 10–20 ml/kg of a regular packed red blood cell transfusion every 2–5 weeks, and the price was 1.05 Chinese Yuan (CNY)/ml-1.525 CNY/ml collected from the bidding price in national centralized tendering procurement in 2021 [19]. Iron chelation mainly included deferoxamine (DFO), deferiprone (DFP), and deferasirox (DFX) at doses as follows: 20–40 mg/kg daily over 5–7 days/ week for DFO, 75–100 mg/kg daily in three divided doses for DFP, and 20–40 mg/kg daily in single doses for DFX. The bidding price and package were as follows: 49.16 CNY/bottle and 500 mg/bottle for DFO; 533 CNY/box and 500 mg * 30 tablets/box for DFP; and 550 CNY/box and 125 mg * 28 tablets/box for DFX [19] (Supplemental file 3). In addition, information on other indicators, including average weight by age [10] and annual survival rate by age, [7] was derived from the published literature (Supplemental file 1).

### Estimation of economic costs

The economic burden per adult β-thalassaemia major patient within a one-year period was estimated from a societal perspective, using a bottom-up method. All costs were presented in 2021 US dollar values using the exchange rate (1 US dollar = 6.3757 CNY) [20]. The economic outcomes are presented as the mean (95% confidence intervals (CI)). The costs with 95% CI in the real world were estimated using the boostrap method, and the mean values ((max + min)/2) with 95% CI (mean value ± 1.96*(max-min)/6) in the clinical guideline were estimated using max and min values.

Annual direct medical costs comprising the costs for annual blood transfusion therapy, iron chelation therapy, and adverse reaction therapy, were extracted from the online survey. Direct medical costs included out-of-pocket (OOP) payments (by patients themselves) and payments covered by health insurers.

Considering patients’ poor therapy adherence, we also calculated annual direct medical costs by weight and cumulative lifetime direct medical costs by weight according to clinical guidelines. Annual direct medical costs by weight were expressed as the sum of the annual transfusion cost and annual iron chelation cost [18]. We did not include the cost for adverse reaction therapy, considering that complications could be negligible if the patient was treated regularly. The annual transfusion cost was calculated by multiplying age-specific weight, blood transfusion per kg per year, and unit cost of blood. Annual iron chelation cost was calculated by multiplying age-specific weight, dosage (number of boxes or bottles) per kg per year, and unit cost of iron chelation per box or bottle. Cumulative lifetime direct medical costs per patient were expressed as the sum of the product of age-specific weight multiplied by direct medical costs per kg per year and survival rate per year in the clinical guidelines. The life expectancy of patients with β-thalassaemia major is assumed to be 50 years old [21].

The annual direct nonmedical cost comprised the annual transportation cost, annual accommodation cost, annual meal and nutrition cost, and annual nursing cost. The annual indirect cost was measured by annual cost due to loss of productivity of patients and caregivers (multiplying total days of lost wages annually and daily lost wages).

### Sensitivity analysis

Univariate sensitivity analyses were conducted to assess the sensitivity of the results to changes in important input parameters, including iron chelation cost, transfusion cost, weight, survival rate, and discount rate. Owing to the large contribution of the cost of iron chelation to the treatment cost, the impact of reducing or increasing the cost by 25% was explored. The impact of doubling or reducing the transfusion cost by 50% was also undertaken [7]. The weight was set to vary by 30%, the survival rate was set to vary by 5%, and the discount rate was set to range from 0–8% [22].

Probabilistic sensitivity analyses were performed to address uncertainties in the input parameters using 2,000 iterations of bootstrap simulation.

## Results

### Characteristics of samples

A total of 75 adult patients with β-thalassaemia major and their primary caregivers (n = 75) from seven provinces were included in this study. The majority of patients were from Guangdong Province (60.0%) and Guangxi Zhuang Autonomous Region (30.7%), and others were from Fujian Province (2.7%), Hunan Province (2.7%), Jiangxi Province (1.3%), Jiangsu Province (1.3%), and Xinjiang Uygur Autonomous Region (1.3%). The mean ages of the patients and caregivers were 24.4 years and 50.6 years, respectively. For adult patients, 50.7% were male, 84.0% were of Han ethnicity, 96.0% were unmarried, 34.7% were employed, 66.7% were diagnosed with comorbidities, 44.0% interrupted blood transfusion therapy, and 38.7% interrupted iron chelation therapy. On average, the disease therapy duration and pretransfusion haemoglobin threshold level were 21.3 years and 76.3 g/L, respectively. For caregivers, the majority were fathers or mothers (89.3%), 25.2% were male, 26.7% were unmarried, and 58.7% had an education level of high school or lower. A minority of caregivers were unemployed (25.3%) or retired (17.3%) (Table 1).

**Table 1:** Characteristics of adult patients with β-thalassaemia major and their caregivers

Categories	Characteristics	Sample
Patient’s demographic	Province, n (%)	
	Guangdong Province	45(60.0)
	Guangxi Zhuang Autonomous Region	23(30.7)
	Other provinces	7(9.3)
	Sex (Male), n (%)	38(50.7)
	Age, years, mean (SD)	24.4(5.5)
	Ethnicity (Han), n (%)	63(84.0)
	Marital status (Unmarried), n (%)	72(96.0)
	Employment status (Employed), n (%)	26(34.7)
Disease and therapy information	Comorbidity (Yes), n (%)	50(66.7)
	Disease therapy duration, years, mean (SD)	21.3(5.0)
	Pretransfusion hemoglobin level (g/L), mean (SD)	76.3(12.3)
	Interruption of blood transfusion therapy in recent year (Yes), n (%)	33(44.0)
	Interruption of iron chelation therapy in recent year (Yes), n (%)	29(38.7)
Caregiver’s demographic	Identity (Father or mother), n (%)	67(89.3)
	Sex (Male), n (%)	19(25.3)
	Age, years, mean (SD)	50.6(9.2)
	Marital status (Unmarried), n (%)	20(26.7)
	Education (Junior high school or lower), n (%)	43(58.7)
	Employment status, n (%)	
	Employed	43(57.3)
	Unemployed	19(25.3)
	Retired	13(17.3)

SD: standard deviation

Other provinces included Fujian Province, Hunan Province, Jiangxi Province, Jiangsu Province, and Xinjiang Uygur Autonomous Region

### Direct medical cost

The mean weight of adult patients with β-thalassaemia major was 56 kg (95% CI: 52–60 kg). The mean annual direct medical cost reported by patients was estimated at $13,478 (95% CI: $11,538-$15,713), including $8,707 (95% CI: $6,734-$10,955) related to the cost for iron chelation therapy, $4,193 (95% CI: $3,595-$4,809) to the cost for blood transfusion therapy, and $785 (95% CI: $473- $1,180) to the cost for adverse reaction therapy (Table 2). The OOP rate was 43.8% in this study.

**Table 2:** Annual direct medical cost of adult patients with β-thalassaemia major

Items	Mean	95% CI	
Weight(kg)	56	52	60
Annual direct medical cost in the real world ($)	13,478	11,538	15,713
Annual cost for blood transfusion therapy	4,193	3,595	4,809
Annual cost for iron chelation therapy	8,707	6,734	10,955
Annual cost for adverse reaction therapy	785	473	1,180
Annual direct medical cost according to the clinical guideline ($)	16,242	9,188	23,296
Annual cost for blood transfusion therapy	3,959	1,999	5,919
Annual cost for iron chelation therapy	12,283	7,190	17,377

CI: confidence interval

According to the clinical guideline, the average annual direct medical cost in patients of the same weight was estimated as $16,242 (95% CI: $9,188-$23,296), comprising a cost for iron chelation therapy of $12,283 (95% CI: $7,190-$17,377) and a cost for blood transfusion therapy of $3,959 (95% CI: $1,999-$5,919) (Table 2).

The undiscounted lifetime direct medical cost for a patient with β-thalassaemia major was estimated to be $518,871 (95% CI: $293,524-$744,217), 75.6% of which was due to the cost of iron chelation therapy;however, when the costs were discounted at a rate of 5%, the lifetime cost was $163,441 (95% CI: $92,458-$234,424). The difference between the undiscounted and discounted totals (8% discount rate), $419,955, demonstrates the impact of discounting (Table 3).

**Table 3:** Cumulative lifetime direct medical cost of adult patients with β-thalassaemia major

Discount rate (%)	Cumulative lifetime direct medical cost ($)			Cumulative lifetime cost for blood transfusion therapy ($)			Cumulative lifetime cost for iron chelation therapy ($)		
	Mean	95% CI		Mean	95% CI		Mean	95% CI	
0	518,871	293,524	744,217	126,470	63,845	189,095	392,400	229,679	555,121
1	397,129	224,655	569,602	96,797	48,865	144,728	300,332	175,790	424,874
2	309,758	175,230	444,286	75,501	38,115	112,887	234,257	137,115	331,399
3	246,066	139,199	352,933	59,977	30,277	89,676	186,090	108,922	263,258
4	198,905	112,520	285,290	48,481	24,474	72,488	150,424	88,046	212,801
5	163,441	92,458	234,424	39,837	20,111	59,564	123,604	72,348	174,860
6	136,367	77,143	195,592	33,238	16,779	49,697	103,129	60,363	145,895
7	115,394	65,278	165,509	28,126	14,199	42,054	87,268	51,079	123,456
8	98,916	55,957	141,875	24,110	12,171	36,049	74,806	43,785	105,827

CI: confidence interval

**Fig. 1:**
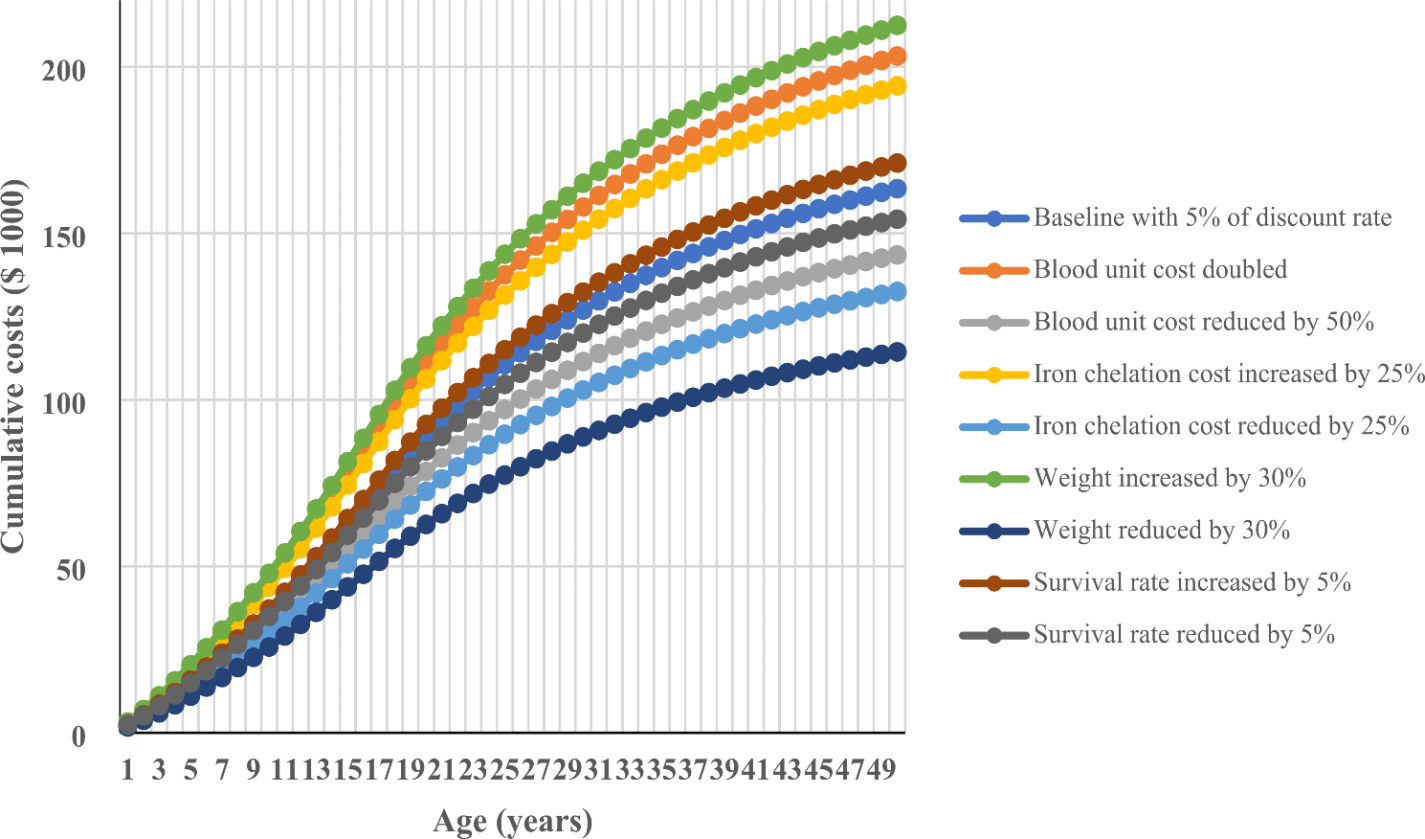
Sensitive analysis for the cumulative lifetime direct medical cost for β-thalassemia major

In the sensitivity analyses, it appeared that a 25% reduction or increase in the iron chelation cost would reduce or increase the lifetime direct medical cost per patient by almost 18.9%. When the cost of a unit of blood was doubled or reduced by 25%, it resulted in a discounted cost of $203,279 or $143,522 for the lifetime direct medical cost, respectively. The discounted lifetime direct medical cost ranged from $114,409 to $212,474 and from $171,118 to $154,269 when body weight or survival rate decreased or increased by 30% and 5%, respectively (Fig. 1).

### Direct nonmedical cost

The annual direct nonmedical cost was estimated as $2,542 (95% CI: $1,356-$4,221), accounting for 12.7% of the total costs per adult patient. The largest share in the annual direct nonmedical cost was transportation costs at $533 (95% CI: $349-$815), followed by meal and nutrition costs at $527 (95% CI: $338-$811), accommodation costs at $353 (95% CI: $211-$534), and nursing costs at $378 (95% CI: $101-$832) (Table 4).

**Table 4:** Annual direct nonmedical cost of adult patients with β-thalassaemia major

Items	Mean	95% CI	
Annual direct nonmedical cost ($)	2,542	1,356	4,221
Annual transportation cost	533	349	815
Annual accommodation cost	353	211	534
Annual meal and nutrition cost	527	338	811
Annual nursing cost	378	101	832

CI: confidence interval

### Indirect cost

The total days of lost wages annually for patients and caregivers due to β-thalassaemia major therapy were 37 days (95% CI: 24–52 days) and 151 days (95% CI: 121–182 days), respectively. Adult patient- and caregiver-reported daily wages were $10 (95% CI: $7-$13) and $24 (95% CI: $19-$28), respectively. The annual indirect cost was estimated at $4,000 (95% CI: $2,596-$5,861), including $295 (95% CI: $176-$433) due to lost working time among patients and $3,905 (95% CI: $2,525-$5,879) due to lost working time among caregivers (Table 5).

**Table 5:** Annual indirect cost of adult patients with β-thalassaemia major

Items	Mean	95% CI	
Annual indirect cost ($)	4,000	2,596	5,861
Annual cost for loss of working time of patient ($)	295	176	433
Total days of lost wage annually for patient (days)	37	24	52
Daily wages for patient ($)	10	7	13
Annual cost for loss of working time of caregiver ($)	3,905	2,525	5,879
Total days of lost wage annually for caregiver (days)	151	121	182
Daily wages for caregiver ($)	24	19	28

CI: confidence interval

## Discussion

Quantifying the economic burden of β-thalassaemia major is an essential step towards developing effective strategies for managing β-thalassaemia major. To our knowledge, this study is the first to estimate the economic costs for adult patients with β-thalassaemia major from a societal perspective, and to estimate the lifetime costs for patients with β-thalassaemia major considering the clinical guideline in mainland China. This study is also the first to compare the differences in direct medical costs between real-world data and clinical guideline data in mainland China.

Our study estimated that the annual direct medical cost, direct non-medical cost, and indirect cost per adult patient with β-thalassaemia major at 56 kg were $13,478, $2,542, and $4,000, respectively. The large difference in annual costs between countries greatly depends on the health care reimbursement system and patients’ treatment compliance. The cost borne by patients and families might be greater due to incomplete universal health care coverage. For example, in the UK, it was estimated that the OOP rate for a β-thalassaemia major patient was only 27.3% [7], which was lower than the level reported in China [23]. With enormous economic costs, many patients in China are experiencing undertreatment according to clinical guidelines, and not all patients receive adequate blood transfusion and iron chelation [2], compared with those in the UK. In addition, the substantial difference might be associated with local medical technology and pricing. In Iran, the total annual direct cost per patient was estimated as €1466 (1 Euro dollar = 1.3936 US dollars), and the indirect cost was €264 in 2009 [15], which was far lower than that in developed countries, such as the UK [14] and US [8]. On the other hand, children were prescribed lower doses of blood transfusion and iron chelation therapy than adults, and costs were substantially associated with patients’ weight and age. In Thai children, it was reported that the annual average treatment cost was $950, the direct nonmedical cost was $274, and the indirect cost was $386 in 2005, which were lower than those costs in adults [11].

We found that direct medical cost was the main driver of total cost among patients with β-thalassaemia major. Our study reported that direct medical cost, direct nonmedical cost, and indirect cost were accounted for approximately 67.3%, 12.7%, and 20.0% of the total cost, respectively, which was similar to another study conducted in Thailand [11]. In addition, our study also found that 64.6% of direct medical cost in the real world and 75.6% according to the clinical guideline were attributable to iron chelation therapy. This finding was similar to some other studies reporting that direct medical cost represented a significant proportion of the total cost, and cost for iron chelation therapy comprised the larger share of the direct medical cost [9].

We found that adult patients with β-thalassaemia major weighing 56 kg were associated with a $2,764 increase in annual direct medical cost according to the clinical guideline, compared with the cost in the real world, which might be attributed to inadequate blood transfusion and irregular iron chelation therapy. The clinical guideline states that a pretransfusion haemoglobin level above 90 g/L is considered normal [18]; however, the mean value was only 76.3 g/L in this study. Inadequate blood transfusion exposes patients with β-thalassaemia major to the effects of chronic anaemia, such as reduced quality of life and long-term complications [2]. In addition, approximately 40% of the patients experienced an interruption of iron chelation therapy because iron chelation was burdensome, painful, and time-consuming. This results in many complications attributable to iron overload, which is toxic to the heart, liver, and endocrine system, eventually resulting in death. When compliance with blood transfusion and iron chelation therapy was good and consistent, 90% of patients survived into their 30s; however, where compliance was poor, fewer than 10% would survive into their 40s [3]. In this study, the maximum age was reported as 43 years old, which was a significant improvement in survival compared to a previous study in China [24]; however, a large gap in survival still existed compared with Hong Kong [25] and Taiwan [26]. Adherence to treatment depended on awareness, which was frequently attributed to the educational level of caregivers [12]; however, our study showed that 58.7% of caregivers had junior high school or lower levels of education.

It was estimated that the undiscounted and discounted (5% discount rate) total lifetime treatment costs were $518,871 and $163,441 in our study, respectively, which were higher than those in Taiwan (undiscounted $363,149, discounted $73,527) [13], but lower than the undiscounted estimates in the UK ($898,851) [14] and Israel ($1,971,380) [27]. The variations in lifetime costs reported by previous studies are due to the different perspectives, time horizons, and patient samples.

For direct nonmedical costs, our study revealed that the annual transportation cost represented 3.3% of the total direct cost, which was consistent with the result in Italy [9]. In addition, the transportation and food costs were the two main components of direct nonmedical cost, which was similar to other studies [9, 28].

Regarding indirect costs, one study conducted in Italy, the UK, and the US reported that the mean number of days spent on disease- management was approximately 3–5 days per month [29], consistent with the results of our study. Compared with caregivers, adult patients were reported to have lower productivity loss despite having to spend more hours in the hospital, which might be because adult patients had a poor earning capacity due to frequent sickness. In addition, patients missed work due to illness, and caregivers missed work to care for patients, including sickness and life care; therefore, caregivers were associated with more total days of lost wages. Some studies reported that caregivers’ burden was high, which was consistent with our results [21, 29].

This study has some limitations. First, the sample size was relatively small. Due to the lack of epidemiological data at a national level and the effects of rather scattered patient residence, COVID-19, and patients’ reluctance to participate, data collection was challenging. However, because β-thalassaemia is a regional rare disease, β-thalassaemia major is only one of three main forms, and surviving adult patients are an even smaller group, the sample size for adult patients with β-thalassaemia major was relatively representative in this study. Second, snowball sampling, as nonprobability sampling, was conducted and responders who voluntarily participate might represent a more compliant and motivated patient in general, owing to the unclear prevalence; therefore, it might not be possible to generalize to all patients. In addition, there was recall bias because the findings relied on responders’ self-reports, sotwo strict quality control interviews were conducted, and the annual direct medical cost and lifetime treatment cost were calculated according to the clinical guideline. Finally, limited published studies or reports were encountered on thalassaemia epidemiology data, age-specific weight, and survival rate; hence, we used data from published studies outside mainland China and expert opinions from clinicians.

## Conclusion

Patients with β-thalassaemia major often encounter a substantial economic burden in mainland China. Direct medical cost was the main driver of the total cost, with blood transfusion and iron chelation therapy as the most expensive components of direct medical cost. It was estimated that the undiscounted and discounted (5% discount rate) total lifetime treatment costs were $518,871 and $163,441, respectively. In addition, using real-world data, adult patients with β-thalassaemia major weighing 56 kg, were associated with a $2,764 increase in the annual direct medical cost estimated based on the clinical guideline; that is, in the real world, the treatment was underutilized compared with the recommendations from the clinical guideline. Efforts must be made to improve patients’ compliance with blood transfusion and iron chelation therapy, facilitate better societal awareness, and help policymakers develop effective strategies to reduce the burden and prevalence of thalassaemia.

### Electronic supplementary material

Below is the link to the electronic supplementary material.

Supplementary Material 1

Supplementary Material 2

Supplementary Material 3

Supplementary Material 4

## Data Availability

All data can be accessed from the sources cited in the manuscript, tables, figures, and supplemental files.

## Abbreviations

UK: United Kingdom
US: United States
COVID-19: Coronavirus
CNY: Chinese Yuan
DFO: Deferoxamine
DFP: Deferiprone
DFX: Deferasirox
CI: Confidence interval
