# Supplementary material for: Economic burden of adult patients with β-thalassaemia major in mainland China
Source: Orphanet J Rare Dis. 2023 Aug 29;18:252. doi: 10.1186/s13023-023-02858-4 (PMC10466866; doi:10.1186/s13023-023-02858-4)
Supplement: Supplementary file 1 — Supplementary Material 1 [file 13023_2023_2858_MOESM1_ESM.docx]

**Supplemental file 1:**

Table S1. Average lifetime direct medical costs for adult patients with β-thalassaemia major

| Age (years) | Weight (kg) | Survival rate (%) | Age (years) | Weight (kg) | Survival rate (%) |
| --- | --- | --- | --- | --- | --- |
| 1 | 8.63 | 99.85 | 26 | 49.37 | 84.42 |
| 2 | 10.37 | 99.69 | 27 | 49.54 | 84.42 |
| 3 | 12.11 | 99.54 | 28 | 49.72 | 84.42 |
| 4 | 13.85 | 99.39 | 29 | 49.89 | 84.42 |
| 5 | 15.59 | 99.24 | 30 | 50.06 | 84.42 |
| 6 | 17.33 | 99.09 | 31 | 50.23 | 82.38 |
| 7 | 19.07 | 98.94 | 32 | 50.4 | 82.38 |
| 8 | 20.81 | 98.79 | 33 | 50.58 | 82.38 |
| 9 | 22.55 | 98.64 | 34 | 50.75 | 82.38 |
| 10 | 24.29 | 97.93 | 35 | 50.92 | 82.38 |
| 11 | 27.98 | 94.38 | 36 | 51.09 | 80.64 |
| 12 | 30.95 | 94.38 | 37 | 51.26 | 80.64 |
| 13 | 33.92 | 94.38 | 38 | 51.44 | 80.64 |
| 14 | 36.89 | 94.38 | 39 | 51.61 | 80.64 |
| 15 | 39.86 | 94.38 | 40 | 51.78 | 80.64 |
| 16 | 42.83 | 90.21 | 41 | 51.95 | 78.84 |
| 17 | 45.8 | 90.21 | 42 | 52.12 | 78.84 |
| 18 | 48.77 | 90.21 | 43 | 52.3 | 78.84 |
| 19 | 48.17 | 90.21 | 44 | 52.47 | 78.84 |
| 20 | 48.34 | 90.21 | 45 | 52.64 | 78.84 |
| 21 | 48.51 | 86.93 | 46 | 52.81 | 76.97 |
| 22 | 48.68 | 86.93 | 47 | 52.98 | 76.97 |
| 23 | 48.86 | 86.93 | 48 | 53.16 | 76.97 |
| 24 | 49.03 | 86.93 | 49 | 53.33 | 76.97 |
| 25 | 49.2 | 86.93 | 50 | 53.5 | 76.97 |

**Supplemental file 2:**

**Translated questionnaire for adult patients with β-thalassaemia major and their caregivers**

**Patient part. Questionnaire for adult patients with β-thalassaemia major**

1. **Patient’s demographics**
2. Province: _____
3. Sex: _____ a. male b. female
4. Date of birth: _____
5. Ethnicity: _____ a. Han b. minority
6. Marital status: _____ a. married b. unmarried
7. Employment status: _____ a. employed b. unemployed
8. **Disease and therapy information**
9. Did you have coexisting diseases? a. yes b. no
10. Which year did you start to treat this disease?
11. Over the past year, did you interrupt blood transfusion therapy? a. yes b. no
12. Over the past year, did you interrupt iron chelation therapy? a. yes b. no
13. Over the past year, what was your hemoglobin level before blood transfusion? (g/L)
14. Over the past year, what was the average weight? (kg)
15. **Direct economic burden (CNY)**
16. Over the past year, how much were the total annual costs for treating β-thalassemia? How much did you pay by yourself?
    1. In which, over the past year, how much were the annual costs of blood transfusion therapy? How much did you pay by yourself?
    2. In which, over the past year, how much were the annual costs of iron chelation therapy? How much did you pay by yourself?
    3. In which, over the past year, how much were the annual costs of adverse reaction therapy due to irregular therapy? How much did you pay by yourself?
17. Over the past year, how much were the monthly direct nonmedical costs including transportation costs, accommodation costs, meal and nutrition costs, and nursing costs?
    1. In which, over the past year, how much were the monthly transportation costs?
    2. In which, over the past year, how much were the monthly accommodation costs?
    3. In which, over the past year, how much were the monthly meal and nutrition costs?
    4. In which, over the past year, how much were the monthly nursing costs?
18. **Indirect economic burden**
19. Over the past year, how much were your total annual incomes? (CNY)
20. Over the past year, how much days of lost working each month for patient? how much days of lost studying each month for patient?

**Caregiver part. Questionnaire for adult patients’ caregivers with β-thalassaemia major**

1. **Caregiver’s demographics**
2. Date of birth: _____
3. Sex: _____ a. male b. female
4. Marital status: _____ a. married b. unmarried
5. Ethnicity: _____ a. Han b. minority
6. Employment status: _____ a. employed b. unemployed c. retired
7. Highest educational level: _____ a. junior high school and below b. above junior high school
8. What is your identity for the patient? a. mother b. father c. others
9. **Caring burden**
10. Over the past year, how many days did you spent to care each month for adult patients with β-thalassemia major?
11. Over the past year, how much were your total annual incomes for caregiver? (CNY)
12. Over the past year, how many days of lost working each month for caregiver?

**Supplemental file 3:**

Table S2. Clinical guidelines for β-thalassaemia in China

| Items | Type | Frequency | Dosage | Bidding price | Package |
| --- | --- | --- | --- | --- | --- |
| Blood transfusion | Red blood cell | 2-5 weeks/time | 10-20ml/kg.time | 1.05-1.525CNY/ml | / |
| Iron chelation | Deferoxamine (DFO) | 5-7 days/week | 20-40mg/kg.day | 49.16CNY/bottle | 500mg/bottle |
|  | Deferiprone (DFP) | 3 times/day | 75-100mg/kg.day | 533 CNY/box | 500mg*30 tablets/box |
|  | Deferasirox (DFX) | 1 time/day | 20-40mg/kg.day | 550 CNY/box | 125mg*28 tablets/box |
