# Supplementary material for: Economic burden of adult patients with β-thalassaemia major in mainland China
Source: Orphanet J Rare Dis. 2023 Aug 29;18:252. doi: 10.1186/s13023-023-02858-4 (PMC10466866; doi:10.1186/s13023-023-02858-4)
Supplement: Supplementary file 3 — Supplementary Material 3 [file 13023_2023_2858_MOESM3_ESM.pdf]

AJE®

Editing Certificate

This document certifies that the manuscript

Economic burden of adult patients with β-thalassemia major in mainland China

prepared by the authors

Xuemei Zhen, Jing Ming, Runqi Zhang, Shuo Zhang, Jing Xie, Baoguo Liu, Zijing Wang, Xiaojie Sun, Lizheng Shi

was edited for proper English language, grammar, punctuation, spelling, and overall style by one or more of the highly qualified native English speaking editors at AJE.

This certificate was issued on **February 10, 2023** and may be verified on the AJE website using the verification code **D5DA-9302-27EE-37A5-2072**.

AJE® EDITING CERTIFIED

Neither the research content nor the authors' intentions were altered in any way during the editing process. Documents receiving this certification should be English-ready for publication; however, the author has the ability to accept or reject our suggestions and changes. To verify the final AJE edited version, please visit our verification page at aje.com/certificate. If you have any questions or concerns about this edited document, please contact AJE at support@aje.com.

AJE provides a range of editing, translation, and manuscript services for researchers and publishers around the world. For more information about our company, services, and partner discounts, please visit aje.com.
