# Supplementary material for: Economic burden of adult patients with β-thalassaemia major in mainland China
Source: Orphanet J Rare Dis. 2023 Aug 29;18:252. doi: 10.1186/s13023-023-02858-4 (PMC10466866; doi:10.1186/s13023-023-02858-4)
Supplement: Supplementary file 4 — Supplementary Material 4 [file 13023_2023_2858_MOESM4_ESM.docx]

Economic burden of adult patients with β-thalassaemia major in mainland China

Xuemei Zhen^1, 2^, Jing Ming^1, 2^, Runqi Zhang^1, 2^, Shuo Zhang^1, 2^, Jing Xie^1, 2^, Baoguo Liu^1, 2^, Zijing Wang^3^, Xiaojie Sun^1, 2*^, Lizheng Shi^4^

^1^ Centre for Health Management and Policy Research, School of Public Health, Cheeloo College of Medicine, Shandong University, No.44 Wenhuaxi Road, Jinan 250012, Shandong, China

^2^ NHC Key Lab of Health Economics and Policy Research (Shandong University), No.44 Wenhuaxi Road, Jinan 250012, Shandong, China

^3^ Beijing New Sunshine Charity Foundation, No.25 Landianchangnan Road, Beijing 100097, China

^4^ Department of Health Policy and Management, School of Public Health and Tropical Medicine, Tulane University, New Orleans, LA 70112, USA

Corresponding author: Xiaojie Sun, E-mail: xiaojiesun@sdu.edu.cn

**Abstract**

**Background**

β-thalassaemia major poses a substantial economic burden, especially in adults. We aimed to estimate the economic burden of adult patients with β-thalassaemia major from a societal perspective using the real-world data. According to the clinical guideline, we also estimated the annual medical costs for patients with the same body weight and calculated the lifetime medical costs over 50 years in mainland China.

**Methods**

This was a retrospective cross-sectional study. An online survey with snowball sampling covering seven provinces was conducted. We extracted patient demographics, caregiver demographics, disease and therapy information, caring burden, and costs for adult patients diagnosed with β-thalassaemia major and their primary caregivers. In the real world, we estimated the annual direct medical cost, direct nonmedical cost, and indirect cost. In addition, we calculated the annual direct medical cost and lifetime direct medical cost by weight with discounted and undiscounted rates according to the clinical guideline.

**Results**

Direct medical costs was the main driver of total cost, with blood transfusion and iron chelation therapy as the most expensive components of direct medical cost. In addition, adult patients with β-thalassaemia major weighing 56kg were associated with an increase of $2,764 in the annual direct medical cost using the real-world data. The undiscounted and discounted (5% discount rate) total lifetime treatment costs were $518,871 and $163,441, respectively.

**Conclusions**

Patients with β-thalassaemia major often encounter a substantial economic burden in mainland China. Efforts must be made to help policymakers develop effective strategies to reduce the burden and pevalence of thalassaemia.

**Keyword**

Economic burden, lifetime cost, adult patient, β-thalassaemia major, China

**Introduction**

β-thalassaemia is a global and regional disease, most highly prevalent in the Mediterranean, Middle East, central Asia, India, and Southern China. The nationwide prevalence of β-thalassaemia is 0.66% in mainland China and as high as 2.21% in high-incidence provinces (such as Guangdong and Guangxi)[1]. β-thalassaemia major, the most severe form, requires regular lifelong blood transfusions and chelation therapy[2]; and poor compliance with blood transfusions and chelation therapy leads to the risks of anaemia and iron overload, resulting in organ damage and complications[3].

Over the last three decades, most patients with β-thalassaemia major were unable to survive to adulthood, mainly because conventional blood transfusions and iron chelation therapy were too burdensome for full adherence. For example, approximately 80% of United Kingdom (UK) patients with β-thalassaemia major died before the age of 45 years[4]. One study with 1,029 patients revealed that adult patients with β-thalassaemia major were rare, and only 3% of patients were 18 years or older in Guangxi,China[5]. In addition, progressive disease results in multiple organ dysfunction, chronic pain, loss of physical fitness, deteriorating quality of life into adulthood, and more challenges than in childhood (such as working and marriage)[6].

The financial burden increases as patients aged and gained weight. Long-term treatment for patients with β-thalassaemia major poses a substantial economic burden on the health care system, patients, and their families[7]. Studies on the economic burden of β-thalassaemia major have been reported in the UK, United States (US), Italy, Iran, Thailand, Taiwan, and India, mainly focusing on children[8-15]. The annual treatment costs of β-thalassaemia major varied across countries and ranged from $950 to $128,062[8, 9, 11, 15]. This massive economic burden is primarily attributed to direct medical cost, and blood transfusions and iron chelation therapy are the most expensive components of direct medical costs[16]. In addition, higher direct medical costs have been found in adult patients than in child patients[17].

Although the direct medical cost in real world has been quite heavy, many patients in China are experiencing undertreatment, and they haven’t received enough blood transfusion and iron chelation owing to poor compliance with the clinical guideline, compared to those in developing countries[2]. Therefore, the economic burden estimation approach based on the real world data may underestimate the medical cost of regular treatments recommended by the clinical guideline. To our knowledge, there is no study of β-thalassaemia major employing adult patient-specific cost data including both direct and indirect costs from a societal perspective, and there is a gap in estimating the lifetime costs for patients with β-thalassaemia major according to the clinical guideline in mainland China.

In this study, we aimed to 1) estimate the economic burden of adult patients with β-thalassaemia major from a societal perspective in the real world, including direct medical costs, direct nonmedical costs, and indirect costs, and 2) estimate the annual medical costs for patients with the same weight and calculate the lifetime medical costs over 50 years, according to the clinical guideline.

**Methods**

**Study design and participants**

This is a retrospective cross-sectional study. An online survey with the “questionnaire star (https://www.wjx.cn)” was conducted between September 1, 2021, and January 31, 2022, because an on-site survey was not feasible during the coronavirus disease 2019 (COVID-19) pandemic. Patients were recruited through the website of the Beijing New Sunshine Charity Foundation and the Thalassaemia Mutual Aid WeChat Group, and patient recommendations by doctors from representative medical institutions in five provinces (Guangdong Province, Guangxi Zhuang Autonomous Region, Yunnan Province, Hainan Province, and Fujian Province) were used as well. Snowball sampling was selected in this study because of the following reasons: first,the prevalence of thalassaemia in different provinces was unclear; second, enough participants were difficult to find, because the patients’ visiting medical institution was not fixed due to the unstable supply of blood and iron chelation, as well as lifetime or irregular therapy; and third, patient’s complete medical cost information were not possible to be collected in one medical institution. In addition, to ensure that there were no missing items, logical errors or irregularities in completion, two strict quality control interviews was conducted via telephone. If necessary, the patients were asked to show hospital expense invoices to provide relatively accurate expense information.

β-thalassaemia major can be diagnosed using blood tests (including complete blood counts and special haemoglobin tests) and genetic testing (e.g. DNA testing)[18]. Adult patients diagnosed with β-thalassaemia major and their caregivers were included in this study. The inclusion criteria for patients were as follows: (a) age ≥18 years old; (b) a diagnosis of β-thalassaemia major before the study; (c) understanding the content of the questionnaire; (d) a primary caregiver who was familiar with the entire treatment process; and (e) completion of two quality control interviews.

**Data collection**

The questionnaire, which included a patient section and a caregiver section, was self-reported by eligible adult patients with β-thalassaemia major and their caregivers. We extracted patient demographics (province, sex, age, ethnicity, marital status, employment status, weight, daily wages), caregiver demographics (sex, age, ethnicity, marital status, employment status, education, identity, daily wage), disease and therapy information (comorbidity, disease therapy duration, pretransfusion haemoglobin level, interruption of blood transfusion therapy or iron chelation therapy, total days of lost wages annually), caring burden (total days of lost wages annually), and costs (direct medical cost, direct nonmedical cost).

Direct medical costs, were calculated by patient weight according to the clinical guideline for the diagnosis and treatment of β-thalassaemia major, which was published by the Subspecialty Group of Haematology, the Society of Paediatrics, and the Chinese Medical Association combined with the Editorial Board, Chinese Journal of Paediatrics in 2017[18], aiming to improve the quality of care through the standardization of management based on evidence in published literature and expert opinion.

According to this clinical guideline, patients with β-thalassaemia major are recommended to receive 10-20 ml/kg of a regular packed red blood cell transfusion every 2-5 weeks, and the price was 1.05 Chinese Yuan (CNY)/ml-1.525 CNY/ml collected from the bidding price in national centralized tendering procurement in 2021[19]. Iron chelation mainly included deferoxamine (DFO), deferiprone (DFP), and deferasirox (DFX) at doses as follows: 20-40 mg/kg daily over 5-7 days/ week for DFO, 75-100 mg/kg daily in three divided doses for DFP, and 20-40 mg/kg daily in single doses for DFX. The bidding price and package were as follows: 49.16 CNY/bottle and 500 mg/bottle for DFO; 533 CNY/box and 500 mg * 30 tablets/box for DFP; and 550 CNY/box and 125 mg * 28 tablets/box for DFX[19] (Supplemental file 3). In addition, information on other indicators, including average weight by age[10] and annual survival rate by age,[7] was derived from the published literature (Supplemental file 1).

**Estimation of economic costs**

The economic burden per adult β-thalassaemia major patient within a one-year period was estimated from a societal perspective, using a bottom-up method. All costs were presented in 2021 US dollar values using the exchange rate (1 US dollar = 6.3757 CNY)[20]. The economic outcomes are presented as the mean (95% confidence intervals (CI)). The costs with 95% CI in the real world were estimated using the boostrap method, and the mean values ((max+min)/2) with 95% CI (mean value ± 1.96*(max-min)/6) in the clinical guideline were estimated using max and min values.

Annual direct medical costs comprising the costs for annual blood transfusion therapy, iron chelation therapy, and adverse reaction therapy, were extracted from the online survey. Direct medical costs included out-of-pocket (OOP) payments (by patients themselves) and payments covered by health insurers.

Considering patients’ poor therapy adherence, we also calculated annual direct medical costs by weight and cumulative lifetime direct medical costs by weight according to clinical guidelines. Annual direct medical costs by weight were expressed as the sum of the annual transfusion cost and annual iron chelation cost[18]. We did not include the cost for adverse reaction therapy, considering that complications could be negligible if the patient was treated regularly. The annual transfusion cost was calculated by multiplying age-specific weight, blood transfusion per kg per year, and unit cost of blood. Annual iron chelation cost was calculated by multiplying age-specific weight, dosage (number of boxes or bottles) per kg per year, and unit cost of iron chelation per box or bottle. Cumulative lifetime direct medical costs per patient were expressed as the sum of the product of age-specific weight multiplied by direct medical costs per kg per year and survival rate per year in the clinical guidelines. The life expectancy of patients with β-thalassaemia major is assumed to be 50 years old [21].

The annual direct nonmedical cost comprised the annual transportation cost, annual accommodation cost, annual meal and nutrition cost, and annual nursing cost. The annual indirect cost was measured by annual cost due to loss of productivity of patients and caregivers (multiplying total days of lost wages annually and daily lost wages).

**Sensitivity analysis**

Univariate sensitivity analyses were conducted to assess the sensitivity of the results to changes in important input parameters, including iron chelation cost, transfusion cost, weight, survival rate, and discount rate. Owing to the large contribution of the cost of iron chelation to the treatment cost, the impact of reducing or increasing the cost by 25% was explored. The impact of doubling or reducing the transfusion cost by 50% was also undertaken[7]. The weight was set to vary by 30%, the survival rate was set to vary by 5%, and the discount rate was set to range from 0% to 8%[22].

Probabilistic sensitivity analyses were performed to address uncertainties in the input parameters using 2,000 iterations of bootstrap simulation.

**Results**

**Characteristics of samples**

A total of 75 adult patients with β-thalassaemia major and their primary caregivers (n=75) from seven provinces were included in this study. The majority of patients were from Guangdong Province (60.0%) and Guangxi Zhuang Autonomous Region (30.7%), and others were from Fujian Province (2.7%), Hunan Province (2.7%), Jiangxi Province (1.3%), Jiangsu Province (1.3%), and Xinjiang Uygur Autonomous Region (1.3%). The mean ages of the patients and caregivers were 24.4 years and 50.6 years, respectively. For adult patients, 50.7% were male, 84.0% were of Han ethnicity, 96.0% were unmarried, 34.7% were employed, 66.7% were diagnosed with comorbidities, 44.0% interrupted blood transfusion therapy, and 38.7% interrupted iron chelation therapy. On average, the disease therapy duration and pretransfusion haemoglobin threshold level were 21.3 years and 76.3 g/L, respectively. For caregivers, the majority were fathers or mothers (89.3%), 25.2% were male, 26.7% were unmarried, and 58.7% had an education level of high school or lower. A minority of caregivers were unemployed (25.3%) or retired (17.3%) (Table 1).

**Direct medical cost**

The mean weight of adult patients with β-thalassaemia major was 56 kg (95% CI: 52-60 kg). The mean annual direct medical cost reported by patients was estimated at $13,478 (95% CI: $11,538-$15,713), including $8,707 (95% CI: $6,734-$10,955) related to the cost for iron chelation therapy, $4,193 (95% CI: $3,595-$4,809) to the cost for blood transfusion therapy, and $785 (95% CI: $473- $1,180) to the cost for adverse reaction therapy (Table 2). The OOP rate was 43.8% in this study.

According to the clinical guideline, the average annual direct medical cost in patients of the same weight was estimated as $16,242 (95% CI: $9,188-$23,296), comprising a cost for iron chelation therapy of $12,283 (95% CI: $7,190-$17,377) and a cost for blood transfusion therapy of $3,959 (95% CI: $1,999-$5,919) (Table 2).

The undiscounted lifetime direct medical cost for a patient with β-thalassaemia major was estimated to be $518,871 (95% CI: $293,524-$744,217), 75.6% of which was due to the cost of iron chelation therapy;however, when the costs were discounted at a rate of 5%, the lifetime cost was $163,441 (95% CI: $92,458-$234,424). The difference between the undiscounted and discounted totals (8% discount rate), $419,955, demonstrates the impact of discounting (Table 3).

In the sensitivity analyses, it appeared that a 25% reduction or increase in the iron chelation cost would reduce or increase the lifetime direct medical cost per patient by almost 18.9%. When the cost of a unit of blood was doubled or reduced by 25%, it resulted in a discounted cost of $203,279 or $143,522 for the lifetime direct medical cost, respectively. The discounted lifetime direct medical cost ranged from $114,409 to $212,474 and from $171,118 to $154,269 when body weight or survival rate decreased or increased by 30% and 5%, respectively (Figure 1).

**Direct nonmedical cost**

The annual direct nonmedical cost was estimated as $2,542 (95% CI: $1,356-$4,221), accounting for 12.7% of the total costs per adult patient. The largest share in the annual direct nonmedical cost was transportation costs at $533 (95% CI: $349-$815), followed by meal and nutrition costs at $527 (95% CI: $338-$811), accommodation costs at $353 (95% CI: $211-$534), and nursing costs at $378 (95% CI: $101-$832) (Table 4).

**Indirect cost**

The total days of lost wages annually for patients and caregivers due to β-thalassaemia major therapy were 37 days (95% CI: 24-52 days) and 151 days (95% CI: 121-182 days), respectively. Adult patient- and caregiver-reported daily wages were $10 (95% CI: $7-$13) and $24 (95% CI: $19-$28), respectively. The annual indirect cost was estimated at $4,000 (95% CI: $2,596-$5,861), including $295 (95% CI: $176-$433) due to lost working time among patients and $3,905 (95% CI: $2,525-$5,879) due to lost working time among caregivers (Table 5).

**Discussion**

Quantifying the economic burden of β-thalassaemia major is an essential step towards developing effective strategies for managing β-thalassaemia major. To our knowledge, this study is the first to estimate the economic costs for adult patients with β-thalassaemia major from a societal perspective, and to estimate the lifetime costs for patients with β-thalassaemia major considering the clinical guideline in mainland China. This study is also the first to compare the differences in direct medical costs between real-world data and clinical guideline data in mainland China.

Our study estimated that the annual direct medical cost, direct non-medical cost, and indirect cost per adult patient with β-thalassaemia major at 56 kg were $13,478, $2,542, and $4,000, respectively. The large difference in annual costs between countries greatly depends on the health care reimbursement system and patients’ treatment compliance. The cost borne by patients and families might be greater due to incomplete universal health care coverage. For example, in the UK, it was estimated that the OOP rate for a β-thalassaemia major patient was only 27.3%[7], which was lower than the level reported in China[23]. With enormous economic costs, many patients in China are experiencing undertreatment according to clinical guidelines, and not all patients receive adequate blood transfusion and iron chelation[2], compared with those in the UK. In addition, the substantial difference might be associated with local medical technology and pricing. In Iran, the total annual direct cost per patient was estimated as €1466 (1 Euro dollar=1.3936 US dollars), and the indirect cost was €264 in 2009[15], which was far lower than that in developed countries, such as the UK[14] and US[8]. On the other hand, children were prescribed lower doses of blood transfusion and iron chelation therapy than adults, and costs were substantially associated with patients’ weight and age. In Thai children, it was reported that the annual average treatment cost was $950, the direct nonmedical cost was $274, and the indirect cost was $386 in 2005, which were lower than those costs in adults[11].

We found that direct medical cost was the main driver of total cost among patients with β-thalassaemia major. Our study reported that direct medical cost, direct nonmedical cost, and indirect cost were accounted for approximately 67.3%, 12.7%, and 20.0% of the total cost, respectively, which was similar to another study conducted in Thailand[11]. In addition, our study also found that 64.6% of direct medical cost in the real world and 75.6% according to the clinical guideline were attributable to iron chelation therapy. This finding was similar to some other studies reporting that direct medical cost represented a significant proportion of the total cost, and cost for iron chelation therapy comprised the larger share of the direct medical cost[9].

We found that adult patients with β-thalassaemia major weighing 56 kg were associated with a $2,764 increase in annual direct medical cost according to the clinical guideline, compared with the cost in the real world, which might be attributed to inadequate blood transfusion and irregular iron chelation therapy. The clinical guideline states that a pretransfusion haemoglobin level above 90 g/L is considered normal[18]; however, the mean value was only 76.3 g/L in this study. Inadequate blood transfusion exposes patients with β-thalassaemia major to the effects of chronic anaemia, such as reduced quality of life and long-term complications[2]. In addition, approximately 40% of the patients experienced an interruption of iron chelation therapy because iron chelation was burdensome, painful, and time-consuming. This results in many complications attributable to iron overload, which is toxic to the heart, liver, and endocrine system, eventually resulting in death. When compliance with blood transfusion and iron chelation therapy was good and consistent, 90% of patients survived into their 30s; however, where compliance was poor, fewer than 10% would survive into their 40s[24]. In this study, the maximum age was reported as 43 years old, which was a significant improvement in survival compared to a previous study in China[25]; however, a large gap in survival still existed compared with Hong Kong[26] and Taiwan[27]. Adherence to treatment depended on awareness, which was frequently attributed to the educational level of caregivers[12]; however, our study showed that 58.7% of caregivers had junior high school or lower levels of education.

It was estimated that the undiscounted and discounted (5% discount rate) total lifetime treatment costs were $518,871 and $163,441 in our study, respectively, which were higher than those in Taiwan (undiscounted $363,149, discounted $73,527)[13], but lower than the undiscounted estimates in the UK ($898,851)[14] and Israel ($1,971,380)[28]. The variations in lifetime costs reported by previous studies are due to the different perspectives, time horizons, and patient samples.

For direct nonmedical costs, our study revealed that the annual transportation cost represented 3.3% of the total direct cost, which was consistent with the result in Italy[9]. In addition, the transportation and food costs were the two main components of direct nonmedical cost, which was similar to other studies[9, 29].

Regarding indirect costs, one study conducted in Italy, the UK, and the US reported that the mean number of days spent on disease- management was approximately 3-5 days per month[30], consistent with the results of our study. Compared with caregivers, adult patients were reported to have lower productivity loss despite having to spend more hours in the hospital, which might be because adult patients had a poor earning capacity due to frequent sickness. In addition, patients missed work due to illness, and caregivers missed work to care for patients, including sickness and life care; therefore, caregivers were associated with more total days of lost wages. Some studies reported that caregivers’ burden was high, which was consistent with our results[21, 30].

This study has some limitations. First, the sample size was relatively small. Due to the lack of epidemiological data at a national level and the effects of rather scattered patient residence, COVID-19, and patients’ reluctance to participate, data collection was challenging. However, because β-thalassaemia is a regional rare disease, β-thalassaemia major is only one of three main forms, and surviving adult patients are an even smaller group, the sample size for adult patients with β-thalassaemia major was relatively representative in this study. Second, snowball sampling, as nonprobability sampling, was conducted and responders who voluntarily participate might represent a more compliant and motivated patient in general, owing to the unclear prevalence; therefore, it might not be possible to generalize to all patients. In addition, there was recall bias because the findings relied on responders’ self-reports, sotwo strict quality control interviews were conducted, and the annual direct medical cost and lifetime treatment cost were calculated according to the clinical guideline. Finally, limited published studies or reports were encountered on thalassaemia epidemiology data, age-specific weight, and survival rate; hence, we used data from published studies outside mainland China and expert opinions from clinicians.

**Conclusion**

Patients with β-thalassaemia major often encounter a substantial economic burden in mainland China. Direct medical cost was the main driver of the total cost, with blood transfusion and iron chelation therapy as the most expensive components of direct medical cost. It was estimated that the undiscounted and discounted (5% discount rate) total lifetime treatment costs were $518,871 and $163,441, respectively. In addition, using real-world data, adult patients with β-thalassaemia major weighing 56 kg, were associated with a $2,764 increase in the annual direct medical cost estimated based on the clinical guideline; that is, in the real world, the treatment was underutilized compared with the recommendations from the clinical guideline. Efforts must be made to improve patients’ compliance with blood transfusion and iron chelation therapy, facilitate better societal awareness, and help policymakers develop effective strategies to reduce the burden and prevalence of thalassaemia.

**Abbreviations**

UK: United Kingdom; US: United States; COVID-19: coronavirus; CNY: Chinese Yuan; DFO: deferoxamine; DFP: deferiprone; DFX: deferasirox; CI: confidence interval.

**Declarations**

**Ethics approval and consent to participate**

This study was approved by the Medical Ethics Committee of the Center for Health Management and Policy Research, Shandong University. All patients and caregivers were voluntary and signed an online informed consent form before collecting data.

**Consent for publication**

Not applicable

**Availability of data and materials**

All data can be accessed from the sources cited in the manuscript, tables, figures, and supplemental files.

**Funding**

The Shandong University Multidisciplinary Research and Innovation Team of Young Scholars (2020QNQT019); The grant project of *economic burden of adult patients with β-thalassaemia* by the New Sunshine Charity Foundation.

**Author contributions**

X.Z. participated in the conception and design of this study, data collection, data analysis, and interpretation of data, drafted and revised the manuscript. J.M., R.Z., S.Z., J.X., and B.L. performed the data analysis, and interpretation of data, drafted and revised the manuscript. Z.W. participated in the conception and design of this study and helped in the data collection. X.S. participated in the conception, design of the study, data collection and interpretation of data, and drafted and revised the manuscript. L.S. participated in the conception and design of the study and helped in revising the manuscript.

**Acknowledgements**

We want to thank the Beijing New Sunshine Charity Foundation, Sun Yat-sen Memorial Hospital, Guangzhou Liwan District People's Hospital, The First Affiliated Hospital of Guangxi Medical University, Guinan Hospital of Guangxi Yulin, Zhongshan Hospital of Xiamen University, Longyan First Hospital of Fujian Medical University, Hospital No. 920 of the People's Liberation Army Joint Security Force, Lincang Hospital of Yunnan Province, Hainan Cancer Hospital, and Hainan Provincial People's Hospital, for assistance in primary data collection.

**Competing interests**

The authors declare no conflict of interest.

**References**

1. Lai K, Huang G, Su L, He Y**.** The prevalence of thalassemia in mainland China: evidence from epidemiological surveys. Sci Rep**.** 2017; 7:920.

2. Shah FT, Sayani F, Trompeter S, Drasar E, Piga A**.** Challenges of blood transfusions in beta-thalassemia. Blood Reviews**.** 2019; 37:100588.

3. Ansari-Moghaddam A, Adineh HA, Zareban I, Mohammadi M, Maghsoodlu M**.** The survival rate of patients with beta-thalassemia major and intermedia and its trends in recent years in Iran. Epidemiology and Health**.** 2018; 40:e2018048.

4. Modell B, Khan M, Darlison M**.** Survival in beta-thalassaemia major in the UK: data from the UK Thalassaemia Register. Lancet**.** 2000; 355:2051-2.

5. Li J, Zhou T, Tang Z, Chen M, Zhou J, Nong P*, et al***.** Investigation on anemia and iron overload in patients with β-thalassemia major in Guangxi. Chinese Journal of Blood Transfusion**.** 2019; 32:183-5.

6. Klaassen RJ, Barrowman N, Merelles-Pulcini M, Vichinsky EP, Sweeters N, Kirby-Allen M*, et al***.** Validation and reliability of a disease-specific quality of life measure (the TranQol) in adults and children with thalassaemia major. Br J Haematol**.** 2014; 164:431-7.

7. Karnon J, Zeuner D, Brown J, Ades AE, Wonke B, Modell B**.** Lifetime treatment costs of beta-thalassaemia major. Clin Lab Haematol**.** 1999; 21:377-85.

8. Weiss M, Parisi JM, Sheth S**.** Clinical and economic burden of regularly transfused adult patients with beta-thalassemia in the United States: a retrospective cohort study using payer claims. American Journal of Hematology**.** 2019; 94:E129-32.

9. Scalone L, Mantovani LG, Krol M, Rofail D, Ravera S, Bisconte MG*, et al***.** Costs, quality of life, treatment satisfaction and compliance in patients with beta-thalassemia major undergoing iron chelation therapy: the ITHACA study. Current Medical Research and Opinion**.** 2008; 24:1905-17.

10. Shafie AA, Wong J, Ibrahim HM, Mohammed NS, Chhabra IK**.** Economic burden in the management of transfusion-dependent thalassaemia patients in Malaysia from a societal perspective. Orphanet Journal of Rare Diseases**.** 2021; 16:157.

11. Riewpaiboon A, Nuchprayoon I, Torcharus K, Indaratna K, Thavorncharoensap M, Ubol BO**.** Economic burden of beta-thalassemia/Hb E and beta-thalassemia major in Thai children. BMC Research Notes**.** 2010; 3:29.

12. Mallik S, Chatterjee C, Mandal PK, Sardar JC, Ghosh P, Manna N**.** Expenditure to treat thalassaemia: an experience at a tertiary care hospital in India. Iranian Journal of Public Health**.** 2010; 39:78-84.

13. Ho WL, Lin KH, Wang JD, Hwang JS, Chung CW, Lin DT*, et al***.** Financial burden of national health insurance for treating patients with transfusion-dependent thalassemia in Taiwan. Bone Marrow Transplantation**.** 2006; 37:569-74.

14. Weidlich D, Kefalas P, Guest JF**.** Healthcare costs and outcomes of managing beta-thalassemia major over 50 years in the United Kingdom. Transfusion**.** 2016; 56:1038-45.

15. Sattari M, Sheykhi D, Nikanfar A, Pourfeizi AH, Nazari M, Dolatkhah R*, et al***.** The financial and social impact of thalassemia and its treatment in Iran. Pharmaceutical Sciences**.** 2012.

16. Betts M, Flight PA, Paramore LC, Tian L, Milenkovic D, Sheth S**.** Systematic literature review of the burden of disease and treatment for transfusion-dependent beta-thalassemia. Clinical Therapeutics**.** 2020; 42:322-37.

17. Alshamsi S, Hamidi S, Narci HO**.** Healthcare resource utilization and direct costs of transfusion-dependent thalassemia patients in Dubai, United Arab Emirates: a retrospective cost-of-illness study. BMC Health Services Research**.** 2022; 22:304.

18. Subspecialty Groups of Hematology, The Society of Pediatrics, Chinese Medical Association Combined with Editorial Board, Chinese Journal of Pediatrics**.** Guideline for diagnosis and treatment of β-thalassemia major (2017). Chinese Journal of Pediatrics**.** 2018; 56:724-9.

19. db. yaozh.com. The bid drug. 2022. https://db.yaozh.com/yaopinzhongbiao?comprehensivesearchcontent. Accessed June 1 2022.

20. Bank of China. Exchange rate. 2021. https://srh.bankofchina.com/search/whpj/search_cn.jsp. Accessed December 31 2021.

21. Beijing AngelMom Charity Foundation. Report on the prevention and treatment of thalassemia in China. 2021. http://www.angelmom.org/content/689. Accessed June 1 2022.

22. Chinese Pharmaceutical Association Pharmacoeconomics Professional. China guidelines for pharmacoeconomic evaluations. 2020. https://www.cpa.org.cn/cpadmn/attached/file/20200929/1601363750111497.pdf. Accessed June 2 2022.

23. New Sunshine Charity Foundation and Shandong University. Economic burden of adult patients with β-thalassemia major in mainland China. 2022. http://www.isun.org/index.php?m=content&c=index&a=show&catid=284&id=1449. Accessed January 11 2023.

24. Ansari-Moghaddam A, Adineh HA, Zareban I, Mohammadi M, Maghsoodlu M**.** The survival rate of patients with beta-thalassemia major and intermedia and its trends in recent years in Iran. Epidemiol Health**.** 2018; 40:e2018048.

25. Yin XL, Wu ZK, He YY, Zhou TH, Zhou YL, Zhang XH**.** Treatment and complications of thalassemia major in Guangxi, Southern China. Pediatric Blood and Cancer**.** 2011; 57:1174-8.

26. Li CK, Luk CW, Ling SC, Chik KW, Yuen HL, Li CK*, et al***.** Morbidity and mortality patterns of thalassaemia major patients in Hong Kong: retrospective study. Hong Kong Medical Journal**.** 2002; 8:255-60.

27. Wu HP, Lin CL, Chang YC, Wu KH, Lei RL, Peng CT*, et al***.** Survival and complication rates in patients with thalassemia major in Taiwan. Pediatric Blood and Cancer**.** 2017; 64:135-8.

28. Koren A, Profeta L, Zalman L, Palmor H, Levin C, Zamir RB*, et al***.** Prevention of beta thalassemia in Northern Israel - a cost-benefit analysis. Mediterranean Journal of Hematology and Infectious Diseases**.** 2014; 6:e2014012.

29. Reed-Embleton H, Arambepola S, Dixon S, Maldonado BN, Premawardhena A, Arambepola M*, et al***.** A cost-of-illness analysis of beta-Thalassaemia major in children in Sri Lanka - experience from a tertiary level teaching hospital. BMC Pediatr**.** 2020; 20:257.

30. Paramore C, Levine L, Bagshaw E, Ouyang C, Kudlac A, Larkin M**.** Patient- and caregiver-reported burden of transfusion-dependent beta-thalassemia measured using a digital application. Patient**.** 2021; 14:197-208.
